# Regulation of myo-miR-24-3p on the Myogenesis and Fiber Type Transformation of Skeletal Muscle

**DOI:** 10.3390/genes15030269

**Published:** 2024-02-21

**Authors:** Danyang Fan, Yilong Yao, Yanwen Liu, Chao Yan, Fanqinyu Li, Shilong Wang, Mei Yu, Bingkun Xie, Zhonglin Tang

**Affiliations:** 1 Key Laboratory of Agricultural Animal Genetics, Breeding and Reproduction of Ministry of Education & Key Lab of Swine Genetics and Breeding of Ministry of Agriculture and Rural Affairs, Huazhong Agricultural University, Wuhan 430070, China; 18339969590@163.com (D.F.); liuyanwen2021666@163.com (Y.L.); yumei@mail.hzau.edu.cn (M.Y.); 2 Kunpeng Institute of Modern Agriculture at Foshan, Agricultural Genomics Institute, Chinese Academy of Agricultural Sciences, Foshan 528226, China; yanchao@caas.cn (C.Y.); lfqyyx@126.com (F.L.); 18236611336@163.com (S.W.); 3 Key Laboratory of Livestock and Poultry Multi-Omics of MARA, Agricultural Genomics Institute at Shenzhen, Chinese Academy of Agricultural Sciences, Shenzhen 518124, China; yaoyilong@caas.cn; 4 Shenzhen Branch, Guangdong Laboratory for Lingnan Modern Agriculture, Agricultural Genomics Institute at Shenzhen, Chinese Academy of Agricultural Sciences, Shenzhen 518124, China; 5 Guangxi Key Laboratory of Livestock Genetic Improvement, Guangxi Institute of Animal Sciences, Nanning 530001, China; bkx@163.com

**Keywords:** porcine, miR-24-3p, skeletal muscle fiber transformation, regeneration

## Abstract

Skeletal muscle plays critical roles in providing a protein source and contributing to meat production. It is well known that microRNAs (miRNAs) exert important effects on various biological processes in muscle, including cell fate determination, muscle fiber morphology, and structure development. However, the role of miRNA in skeletal muscle development remains incompletely understood. In this study, we observed a critical miRNA, miR-24-3p, which exhibited higher expression levels in Tongcheng (obese-type) pigs compared to Landrace (lean-type) pigs. Furthermore, we found that miR-24-3p was highly expressed in the dorsal muscle of pigs and the quadriceps muscle of mice. Functionally, miR-24-3p was found to inhibit proliferation and promote differentiation in muscle cells. Additionally, miR-24-3p was shown to facilitate the conversion of slow muscle fibers to fast muscle fibers and influence the expression of *GLUT4*, a glucose transporter. Moreover, in a mouse model of skeletal muscle injury, we demonstrated that overexpression of miR-24-3p promoted rapid myogenesis and contributed to skeletal muscle regeneration. Furthermore, miR-24-3p was found to regulate the expression of target genes, including *Nek4*, *Pim1*, *Nlk*, *Pskh1*, and *Mapk14*. Collectively, our findings provide evidence that miR-24-3p plays a regulatory role in myogenesis and fiber type conversion. These findings contribute to our understanding of human muscle health and have implications for improving meat production traits in livestock.

## 1. Introduction

Skeletal muscle plays a crucial role in physiological processes and is one of the most abundant tissues in the body. In addition to maintaining normal movement, skeletal muscle serves as the largest metabolic organ, regulating energy metabolism by secreting regulatory factors and communicating with distal organs [1,2]. However, it is sensitive to injury or disease, either from direct wounds or indirect causes such as congenital genetic defects, which can ultimately impact health [3,4,5]. If not repaired in time, the damage may lead to reduced muscle mass and impaired exercise capacity. During the process of myogenesis and skeletal muscle regeneration, muscle stem cells differentiate and fuse together to form multinucleated myotubes [6,7,8,9]. Mammals generally have two types of muscle fibers: fast-twitch fibers and slow-twitch fibers [10,11,12,13,14]. Both mice and humans have a certain degree of plasticity in their muscle fiber types, meaning that under specific environmental stimuli or training, muscle fibers can undergo transformation or adaptive changes [15,16]. However, there are certain differences in muscle fiber types between humans and mice. Research has shown that the proportion of slow-twitch fibers in mouse muscles is usually higher than in humans. Humans appear to have no type IIb fibers expressing *MYH4*, but more type IIX [2,17]. Despite the differences in muscle fiber types between humans and mice, the basic characteristics of mouse muscle fibers are similar to those of humans [18,19]. Therefore, studying mice can help reveal some fundamental physiological features of human muscles. The differences in endurance, number of satellite cells, and mode of energy metabolism between muscle fiber types determine muscle function. Additionally, the composition of myofiber types and skeletal muscle growth and development significantly affect livestock and poultry meat yield and quality [20,21]. Therefore, a thorough analysis of the molecular regulatory networks that regulate myogenesis and changes in myofiber type composition is crucial for promoting human skeletal muscle health and improving meat production traits in livestock.

Numerous studies have shown that skeletal muscle development is regulated by multi-modal, multi-level, and multi-dimensional molecular targets and complex genetic mechanisms [22,23]. As crucial regulatory factors, miRNAs play a significant role in various biological processes. The study has shown that certain key miRNAs influence muscle development, affecting the size and type of muscle fibers during prenatal and postnatal growth [24]. Additionally, highly expressed skeletal muscle-specific miRNAs, such as miR-1 and miR-206, have similar seed sequences and perform similar functions in regulating myogenesis [25,26]. During skeletal muscle cell differentiation, miR-1 promotes muscle cell differentiation and myofibrillar production by targeting *PAX3*, *PAX7*, *HDAC4*, *CX43*, *CNN3*, *SFRP1*, *YY1*, and *NOTCH3* genes [27,28]. Furthermore, *PAX3*, *PAX7*, *HDAC4*, *CX43*, and *NOTCH3* have been shown to be targets of miR-206 [28,29]. Additionally, miR-1 and miR-206 affect skeletal muscle regeneration by regulating skeletal muscle satellite cell activation [30,31,32]. Moreover, miRNAs encoded by myofiber type marker genes regulate changes in myofiber type composition. For instance, miR-499 and miR-208b, encoded by *MYH7B*, drive the generation of slow contractile fibers by down-regulating transcriptional repressors of slow muscle marker genes [33]. Furthermore, there is evidence to support that miR-499-5p can promote the formation of oxidative myofibers by down-regulating the expression of pSox6 [34], and miR-499 and miR-208b can target *SOX6* and *PURB* and rescue the deficiency of slow myogenesis in mice [35]. Research has also indicated that miR-152 can enhance the formation of slow-twitch muscle fibers and contribute to skeletal muscle development by targeting *UCP3* [36]. In addition, a study has demonstrated that miR-27a-3p and miR-142-3p play a regulatory role in skeletal muscle lipid utilization [37]. Our group previously found that miR-143a, miR-133a, miR-743a-5p, and miR-200c can regulate myogenesis and skeletal muscle regeneration by regulating skeletal muscle cell proliferation, differentiation, and migration [38,39,40,41]. However, the roles of miRNA in myogenesis, skeletal muscle regeneration, and muscle type fiber transformation are not fully understood.

In this study, we explored the importance of miR-24-3p, an essential miRNA, in regulating myogenesis and skeletal muscle fiber type transformation. Based on the porcine skeletal muscle differentially expressed miRNA map constructed in our previous study, miR-24-3p was found to be differentially expressed in the skeletal muscle embryonic and postnatal stages. Additionally, we observed that miR-24-3p in skeletal muscle was associated with myofiber type transformation and skeletal muscle injury regeneration in vivo and in vitro. Overexpression of miR-24-3p promoted skeletal muscle regeneration and fast myofiber production. Furthermore, we demonstrated that miR-24-3p can regulate the downstream mRNA expression of genes. This study serves as a valuable resource for animal breeding and contributes to our understanding of skeletal muscle biology and related diseases.

## 2. Materials and Methods

### 2.1. Experiment Animals

The experiment utilized male SPF C57BL/6 mice purchased from Guangdong Vital River Laboratory Animal Technology Co., Ltd. (Guangzhou, China), which were 4 weeks and 8 weeks old and had a body weight of 25–30 g. All mice were kept under controlled conditions, including a constant temperature of 26 °C, 60% relative humidity, a 12-h light–dark cycle, and ad libitum access to food and water. Male Landrace piglets (1 week old) were purchased from Guanghui Agriculture and Animal Husbandry Group (Foshan, China).

### 2.2. Isolation and Culture of Porcine Primary Myoblasts

To obtain primary myoblast cells for our study, we collected hind leg muscles from piglets that were less than one week old. In simple terms, the skeletal muscles were collected and digested with 300 U/mL type II collagenase (Gibco, Grand Island, NY, USA) at 37 °C for 30 min. The digestion was then terminated by adding high glucose culture medium (DMEM, Grand Island, NY, USA) supplemented with 10% fetal bovine serum (FBS, Grand Island, NY, USA) and 2% antibiotics, followed by filtration through 40, 70, and 100 μm filters to remove tissue debris. The cell pellet was resuspended and cultured in RPMI-1640 medium (Gibco, Grand Island, NY, USA), and purified satellite cells were transferred to culture dishes coated with Matrigel (BD Biosciences, San Jose, CA, USA) for proliferation. When the primary myoblast cells reached 90% confluency, differentiation was induced using 5% horse serum (HS, Grand Island, NY, USA) in DMEM.

### 2.3. Cell Culture and Transfection

Porcine skeletal muscle cells (BIOSPECIES-0017a) were procured from Guangzhou Suyan Biotechnology Co., Ltd. (Guangzhou, China). These cells were cultured in Dulbecco’s Modified Eagle’s Medium (DMEM, Gibco, Grand Island, NY, USA) supplemented with 20% fetal bovine serum (FBS, Gibco, Grand Island, NY, USA) and 1% penicillin-streptomycin (PS, Thermo Scientific, Waltham, MA, USA). The cells were maintained and cultured in a humidified incubator at 37 °C with 5% CO_2_. Following the instructions provided by the JetPRIME transfection reagent (Polyplus-transfection, Illkirch, France), we transfected inhibitors and mimics into porcine skeletal muscle cells to achieve knockdown and overexpression of miR-24-3p. To serve as blank controls, we included inhibitor NC and mimics NC.

We procured C2C12 myoblasts and HEK293T cells from the American Type Culture Collection (ATCC, Manassas, VA, USA) for in vitro experiments. C2C12 myoblasts and HEK293T cells were cultured in Dulbecco’s Modified Eagle’s Medium (DMEM, Gibco, Grand Island, NY, USA) supplemented with 10% fetal bovine serum (FBS, Gibco, Grand Island, NY, USA), 1% penicillin, 100 mg/mL streptomycin, and 1% glutamine (PS, Thermo Scientific, Waltham, MA, USA). The cells were maintained in a humidified incubator at 37 °C with 5% CO_2_.

Cell transfection in this study was performed according to the instructions provided by JetPRIME (Polyplus-transfection, Illkirch, France). To increase the cell survival rate by 25–30% after transfection, we added 2% FBS to the transfection medium. After transfection for 6 h, the medium was replaced with a complete medium containing 10% FBS and 1% PS.

### 2.4. Inducing Cell Differentiation

Cell differentiation was induced using Dulbecco’s Modified Eagle’s Medium (DMEM, Gibco, Grand Island, NY, USA) supplemented with 2% horse serum (Gibco, Grand Island, NY, USA) when the cell densities of the primary myoblasts from porcine skeletal myoblasts (PSMC) and C2C12 myoblasts had reached 90% to 95% 6 h after transfection. Cell samples were collected 48–72 h after the induction of differentiation.

### 2.5. Construction of the Skeletal Muscle Injury Model

Male SPF C57BL/6 mice that were 8 weeks old and had similar body weight were obtained from a commercial supplier (Weitonglihua, Guangzhou, China). To establish a regeneration model in the mice, a total volume of 20 μL of 10 nM cardiotoxin (CTX, Tokyo, Japan, MCE, Monmouth Junction, NJ, USA) was injected into the tibialis anterior muscle, causing skeletal muscle injury. This procedure was performed in accordance with a previously described protocol [27,29]. The mice were housed under identical environmental conditions. Muscle samples were collected at 0d, 1d, 3d, 5d, 7d, and 14d post-injury to examine mRNA and protein expression levels, as well as tissue cross-section.

Additionally, a batch of mice with SPF was selected. We injected 100 μL of AAV9-miR-24-3p (packgene, Guangzhou, China) at a titer of 10^9^ GC/mL into the left tibialis anterior muscle of each mouse and concurrently injected the same dose and titer of AAV9-NC (packgene, Guangzhou, China) into the right tibialis anterior muscle as a control. The mice were housed under indentical environmental conditions. After the 30th day, a mouse was randomly selected for dissection and sampling to quickly detect the overexpression effect of miR-24-3p. On the same day, the batch of mice was injected with 20 μL of 10 nM CTX into the tibialis anterior muscle to establish the skeletal muscle injury and regeneration. Samples were collected at different time points after injection (1d, 3d, 5d, 7d, and 14d) and analyzed for mRNA and protein expression levels and tissue sections to compare the effect of skeletal muscle regeneration in mice.

### 2.6. CCK-8 Assay

The CCK-8 assay was conducted on primary porcine skeletal muscle cells and C2C12 myoblasts, which were seeded in a 96-well plate for transfection. Cell proliferation was detected using the Cell Counting Kit-8 (Beyotime Biotechnology, Shanghai, China) at 0 h, 24 h, 36 h, 48 h, and 72 h after transfection. To perform the assay, a mixture of CCK-8 reagent and complete culture medium was prepared at a 1:9 ratio. This mixture was then added to the 96-well plate, followed by incubation at 37 °C for 40 h. The absorbance of the samples at 450 nm was measured using a microplate reader, and the resulting optical density (OD) values were used to construct a growth curve.

### 2.7. 5-Ethynyl-2′-Deoxyuridine Staining (EdU Staining)

The cells were cultured in 12-well plates until they reached approximately 50% confluence. Subsequently, the cells were transfected with plasmids, siRNA, miRNA mimics, or corresponding control molecules. After 24 h of transfection, the assay was conducted as per the instructions provided in the EdU kit (Beyotime Biotechnology, Shanghai, China). To observe the number of EdU-stained cells, a fluorescence confocal microscope (Nikon, A1HD25, Tokyo, Japan) was utilized. Three random areas were selected.

### 2.8. Real-Time Quantitative PCR (qRT-PCR)

Additionally, according to the provided instructions, Fast ChamQ Universal SYBR qPCR Master Mix (Vazyme, Nanjing, China) was utilized for mRNA quantitative real-time PCR (qRT-PCR). In brief, the total RNA from both cells and tissues was extracted using Trizol (Invitrogen, Carlsbad, CA, USA), and the cytoplasmic and nuclear RNA fractions were extracted by the Cytoplasmic Nuclear RNA Extraction Kit (Norgen Biotek, Solana Beach, CA, USA). To generate cDNA from mRNA, the HiScript III 1st Strand cDNA Synthesis kit (+gDNA wiper) (Vazyme, Nanjing, China) was implemented following the manufacturer’s instructions. For cDNA synthesis from miRNA, the miRNA 1st Strand cDNA Synthesis kit (by stem-loop) (Vazyme, Nanjing, China) was employed in accordance with the manufacturer’s instructions. According to the provided instructions, the mRNA quantitative real-time PCR (qRT-PCR) was carried out using Fast ChamQ Universal SYBR qPCR Master Mix (Vazyme, Nanjing, China). The amount of RNA used for reverse transcription was 1000 ng, and the resulting cDNA was diluted with 20 µL of water, and 1 µL of cDNA was added to 10 µL of the reaction system for qRT-PCR. The formula we utilized to calculate the fluorescence amplification efficiency was as follows: E = (10^−1/slope^ − 1) × 100%. Amplification efficiencies were calculated to be 90 to 100%. In this formula, E represents the fluorescence quantitative amplification efficiency, and slope refers to the slope of the fluorescence curve. For the calculation of relative expression levels, the 2^−ΔΔCT^ method was employed. *NEAT1* is a well-known long non-coding RNA (lncRNA) that predominantly localizes to the nucleus. *GAPDH* (Glyceraldehyde 3-phosphate dehydrogenase) is a gene that is primarily enriched in the cytoplasm [42]. Primer sequences are listed in Table S1.

### 2.9. Western Blot

Protein samples were extracted from treated cells or tissues by protein lysis buffer (Thermo Fisher, Waltham, MA, USA). The lysate consisted of RIPA buffer (Beyotime Biotechnology, Shanghai, China) and PMSF (Solarbio, Beijing, China). The concentration of the resulting proteins was determined using the BCA kit from Beyotime. In order to denature the protein samples, sodium dodecyl sulfate (SDS, CWBIO, Beijing, China) was added, and the samples were heated at 100 °C for 20 min. Next, 10% and 12% sodium dodecyl sulfate-polyacrylamide gel electrophoresis (SDS-PAGE) gels (EpiZyme, Shanghai, China) were chosen with a sample volume of 10 µL. Following electrophoresis, the protein bands were transferred onto a 0.45 μm hybridized nitrocellulose filter membrane (NC) (Merck & Co., Rahway, NJ, USA) and sealed with 5% skimmed milk powder. The membrane was then incubated with primary and secondary antibodies. Primary antibodies containing KI67 (358 kDa, 1:1000, Bioss bs-23102R, Beijing, China), PCNA (34 kDa, 1:1000, Affinity Biosciences AF0239, Liyang, China), CDK4 (34 kDa, 1:1000, Cell Signaling Technology, 1290S, Danvers, MA, USA), CYCLIND2 (50 kDa, 1:1000, Affinity Biosciences AF5410, Liyang, China), Myh4 (224kd, 1:1000, Proteintech, 20140-1-AP, Wuhan, China), Myh7 (224kd, 1:1000, Proteintech, 22280-1-AP, Wuhan, China), MyoG (25kd, 1:1000, Proteintech, Ag25081, Wuhan, China), MYHC (223kd, 1:1000, Proteintech, 22281-1-AP, Wuhan, China), and GAPDH (37kd, Abcam ab9482, Cambridge, UK) were diluted with 1× Tween (TBST) buffer (EpiZyme, Shanghai, China) in accordance with the manufacturer’s instructions. Secondary antibodies were from rabbits and mice. The targeted and referenced proteins were detected using the Gel Doc XR System (Bio-Rad, Hercules, CA, USA) according to the manufacturer’s instructions. Gray scale values of protein bands were analyzed using ImageJ 1.52i software (NIH, Bethesda, MD, USA).

### 2.10. Muscle Tissue Section and Staining

After anesthetizing the mice, the tibialis anterior and gastrocnemius muscles were dissected. The muscles were embedded in OCT compound (Sakura, Torrance, CA, USA) and rapidly frozen by liquid nitrogen fixation. Then, frozen sections (10 μm) were obtained using a cryostat. The sections were fixed with 4% paraformaldehyde for 5–8 min and then stained with H&E staining reagents (Solarbio H&E staining kit, Beijing, China) following the manufacturer’s instructions. For tissue immunofluorescence staining, the sections were fixed with 4% paraformaldehyde, permeabilized with 0.5% Triton-100 (Thermo Fisher, Waltham, MA, USA) for 10 min at room temperature, and rinsed with PBS (Gibco, Gaithersburg, MD, USA). Then, the sections were blocked with 5% BSA (Merck, Rahway, NJ, USA) at 37 °C for 1 h. Subsequently, they were incubated with primary antibodies for 1 h at room temperature or 3 h at room temperature. After washing with PBS, the sections were incubated with fluorescent secondary antibodies at room temperature in the dark for 1–2 h. Finally, DAPI (Thermo Fisher, Waltham, MA, USA) staining was added to stain the nuclei. The stained sections were observed and photographed under a fluorescence microscope. The procedure for cell immunofluorescence staining is similar to that for tissue immunofluorescence staining.

### 2.11. Luciferase Activity Assay

HEK293T cells were cultured in a 24-well plate and incubated until reaching 80% confluency. Then, the cells were co-transfected with miRNA mimics or their controls, along with wild-type and mutant dual luciferase reporter vectors containing the predicted target genes. Cells were collected 48 h after transfection. Following the instructions for the Dual Luciferase Reporter Assay System kit, the fluorescence ratio of firefly and Renilla luciferase was measured using a luminometer from Promega (Promega, Madison, WI, USA).

### 2.12. Target Gene Prediction

Using the online databases miRDB (http://mirdb.org/), TargetScan (http://www.targetscan.org/vert_71/), and miRWalk (http://mirwalk.umm.uni-heidelberg.de/), prediction of murine-derived miRNA target genes was performed. We accessed these websites on 15–20 October 2022.

### 2.13. Statistical Analysis

Statistical analyses were performed using GraphPad Prism 9.0 software. Each experiment was repeated a minimum of three times. The significance of differences between groups was determined using a *t*-test and an ANOVA. Statistical significance was assessed with a *p*-value less than 0.05 considered statistically significant (* *p* < 0.05), a *p*-value less than 0.01 considered highly statistically significant (** *p* < 0.01), and a *p*-value less than 0.001 considered more highly statistically significant (*** *p* < 0.001). Data were expressed as mean ± S.E.M.

## 3. Results

### 3.1. miR-24-3p Play a Role in Regulation of Myogenesis and Muscle Formation in Porcine Skeletal Muscle

**Figure 1:**
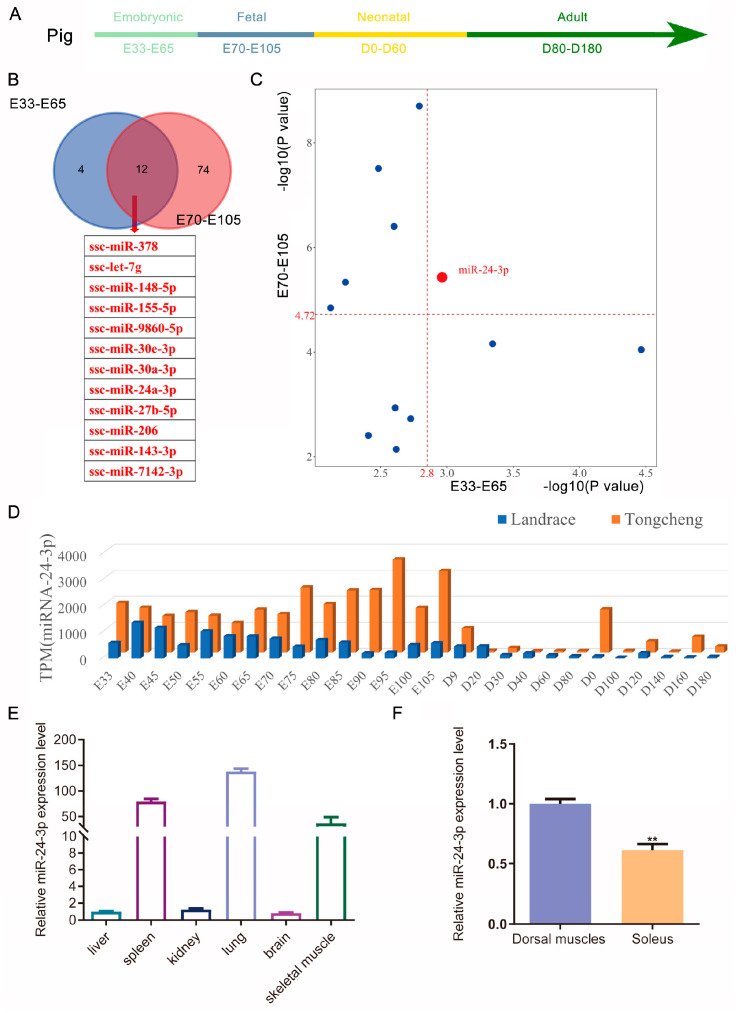
miR-24-3p emerges as a potential regulatory factor involved in skeletal muscle development. (**A**) Diagram of the different developmental periods of the pig. (**B**) A Venn diagram was constructed to illustrate the differentially expressed miRNAs between the embryonic and postnatal stages of Tongcheng and Landrace pigs. (**C**) Scatter plots were generated to visualize the differentially expressed miRNAs that were shared between the embryonic and postnatal stages of skeletal muscle development. (**D**) Expression patterns of miR-24-3p during the embryonic and postnatal stages in Tongcheng and Landrace pigs. (**E**) The miR-24-3p expression pattern in different tissues. (**F**) The miR-24-3p expression in different types of skeletal muscle. The data were presented as mean ± S.E.M. and analyzed for statistical differences between groups using unpaired two-tailed *t*-tests. ** *p* < 0.01.

By further analyzing the miRNA data from 27 time points during porcine skeletal muscle development in our previous study [43], we discovered a specific group of 12 miRNAs during the E33–E65 and E70–E105 stages (Figure 1A,B). Upon further analysis of the thresholds (mean values) in the two developmental periods, we observed a significant difference in the expression of miR-24-3p during both the embryonic (E33–E65) and fetal (E70–E105) stages (Figure 1C). Additionally, miR-24-3p exhibited higher expression levels in Tongcheng (TC) pigs compared to Landrace (LD) pigs (Figure 1D). Furthermore, our analysis revealed that miR-24-3p exhibited a high expression level in the skeletal muscle of both pigs and mice, with expression in fast-contracting muscles being significantly higher than in slow-contracting muscles (Figure 1E,F and Figure S1A–C). These findings suggest that miR-24-3p may play a crucial role in myogenesis and skeletal muscle formation.

### 3.2. miR-24-3p Inhibits Skeletal Muscle Cell Proliferation

To explore the role of miR-24-3p in myogenesis, we performed experiments to examine its impact on PSMCs. Specifically, we evaluated the effect of both knockdown and overexpression of miR-24-3p on PSMC proliferation using a CCK-8 assay. The results of these assays showed that overexpression of miR-24-3p significantly inhibited cell proliferation, whereas knockdown of miR-24-3p promoted cell proliferation (Figure S2A,B). Furthermore, we conducted qRT-PCR and Western blot assays to validate that miR-24-3p does influence PSMC proliferation. Specifically, knockdown of miR-24-3p resulted in enhanced expression of proliferation markers (*KI67*, *PCNA*, *CDK4*, *Cyclin A*, and *Cyclin D2*), while overexpression of miR-24-3p exerted the opposite effects on the expression of these proliferation markers (Figure S2C–E).

**Figure 2:**
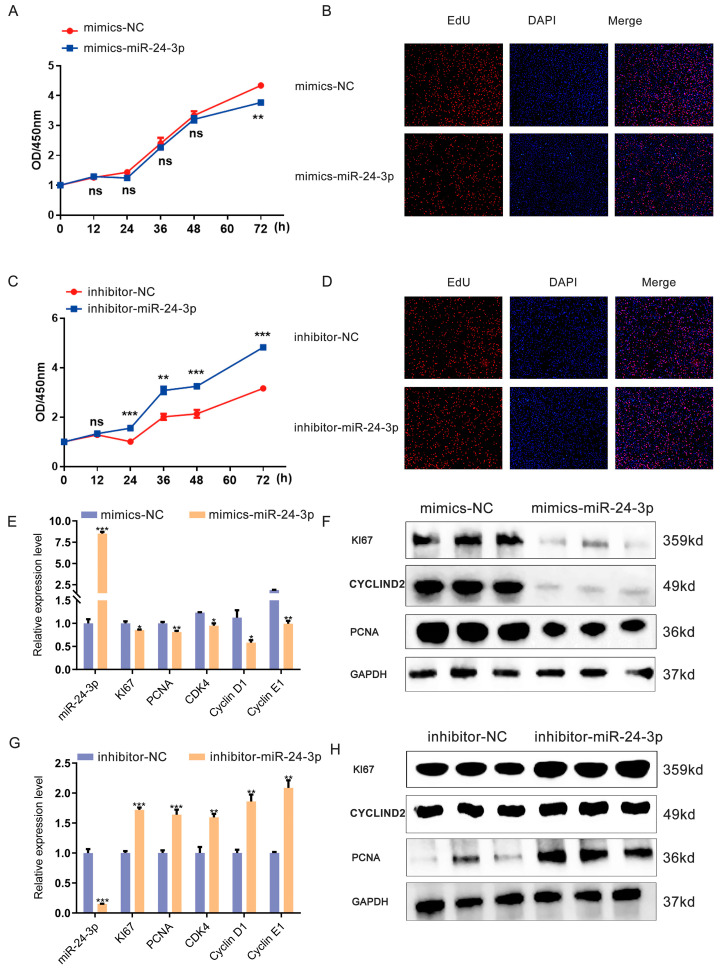
Effects of miR-24-3p on C2C12 myoblast proliferation. (**A**–**D**) The proliferation of C2C12 myoblasts was analyzed after overexpression, and the knockdown of miR-24-3p was determined by a CCK-8 assay and EdU staining. (**E**,**F**) qRT-PCR was used to analyze proliferation marker (*KI67*, *PCNA*, *CDK4*, *Cyclin D1*, and *Cyclin E1*) expression after miR-24-3p overexpression and knockdown. (**G**,**H**) Western blot was used to analyze proliferation marker (KI67, PCNA, and Cyclin D1) expression after miR-24-3p overexpression and knockdown. The data were presented as mean ± S.E.M. and analyzed for statistical differences between groups using unpaired two-tailed *t*-tests. * *p* < 0.05, ** *p* < 0.01, *** *p* < 0.001, ns (not significant).

Moreover, we conducted a sequence analysis to investigate the conservation of miR-24-3p across different species. Interestingly, our findings revealed that miR-24-3p exhibits conservation in pigs and mice (Figure S3). Hence, we conducted additional experiments to gain a deeper understanding of the regulatory role of miR-24-3p in C2C12 myoblasts. Through CCK-8 assay and EdU staining, we observed that overexpression of miR-24-3p significantly decreased the number of proliferating C2C12 myoblasts as well as the number of EdU-positive cells when compared to the mimics NC group (Figure 2A,B). Conversely, inhibitors of miR-24-3p led to the promotion of C2C12 myoblast proliferation (Figure 2C,D). The qRT-PCR results revealed significant down-regulation of the expression levels of C2C12 myoblast proliferation markers, including *KI67*, *PCNA*, *CDK4*, *Cyclin D1*, and *Cyclin E1*, upon overexpression of miR-24-3p. Conversely, knockdown of miR-24-3p results in an up-regulation of these marker expression levels (Figure 2E,F). Furthermore, the protein expression of C2C12 myoblast proliferation markers exhibited a consistent pattern with mRNA expression upon both knockdown and overexpression of miR-24-3p (Figure 2G,H). These findings collectively indicate that miR-24-3p plays a role in regulating cell proliferation in pigs and mice.

### 3.3. miR-24-3p Plays a Significant Role in Promoting Skeletal Muscle Cell Differentiation

**Figure 3:**
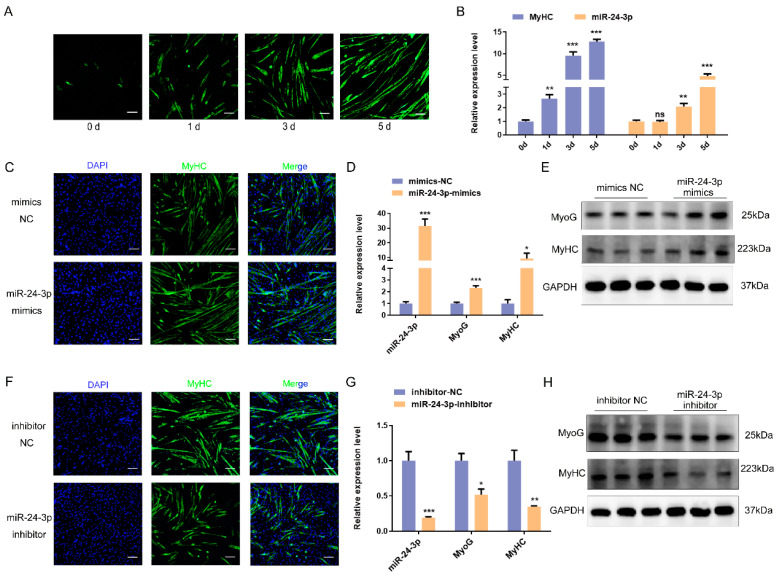
Effects of miR-24-3p on C2C12 myoblast differentiation. (**A**) C2C12 myoblasts were induced to differentiate for different lengths of time (0d, 1d, 3d, and 5d). Scale bar: 50 μm. (**B**) qRT-PCR was used to analyze the expression pattern of miR-24-3p during C2C12 myoblast differentiation. (**C**) Immunofluorescence staining was used to analyze the effect of miR-24-3p overexpression on C2C12 myoblast differentiation. Scale bar: 50 μm. (**D**,**E**) qRT-PCR and Western blot were used to analyze the effect of miR-24-3p overexpression on C2C12 myoblast differentiation marker expression. (**F**) Immunofluorescence staining was used to analyze the effect of miR-24-3p knockdown on C2C12 myoblast differentiation. Scale bar: 50 μm. (**G**,**H**) qRT-PCR and Western blot were used to analyze the effect of miR-24-3p knockdown on C2C12 myoblast differentiation marker expression. The data were presented as mean ± S.E.M. and analyzed for statistical differences between groups using unpaired two-tailed *t*-tests. * *p* < 0.05, ** *p* < 0.01, *** *p* < 0.001, ns (not significant).

Additionally, we analyzed the impact of miR-24-3p on cell differentiation. In vitro, we induced the differentiation of PSMCs and C2C12 myoblasts using 5% and 2% horse serum, respectively. We then examined the expression pattern of miR-24-3p at different time points (0, 1, 3, and 5 days) during cell differentiation (Figure 3A and Figure S4A). The qRT-PCR assay revealed that miR-24-3p expression during the differentiation of PSMCs and C2C12 myoblasts exhibited a similar trend to that of *MyHC* expression, with miR-24-3p expression gradually increasing (Figure 3B and Figure S4B). These results suggest that miR-24-3p may play a regulatory role in skeletal muscle cell differentiation in both pigs and mice.

Subsequently, we conducted additional experiments using qRT-PCR and Western blot to further investigate the effects of miR-24-3p overexpression and knockdown on PSMC differentiation. The qRT-PCR results demonstrated that overexpression of miR-24-3p led to an up-regulation in the expression of cell differentiation markers (*MyoD*, *MyoG*, and *MyHC*), whereas interference with miR-24-3p resulted in down-regulation of these three markers (Figure S4C,D). Furthermore, Western blot analysis revealed increased expression levels of MyoG and MyHC in the group transfected with miR-24-3p mimics compared to the mimics NC group. Conversely, the expression levels of *MyoG* and *MyHC* were reduced after miR-24a-3p knockdown (Figure S4E,G).

Similar to the results observed in PSMCs, overexpression of miR-24-3p in C2C12 myoblasts promoted cell differentiation and up-regulated mRNA and protein expression of differentiation markers (Figure 3C–E). Conversely, knockdown of miR-24-3p inhibited C2C12 myoblast differentiation and down-regulated mRNA and protein expression of differentiation markers (Figure 3F–H). These findings provide further evidence that miR-24-3p can regulate skeletal muscle cell differentiation.

### 3.4. miR-24-3p Regulates Skeletal Muscle Fiber Type Transformation

**Figure 4:**
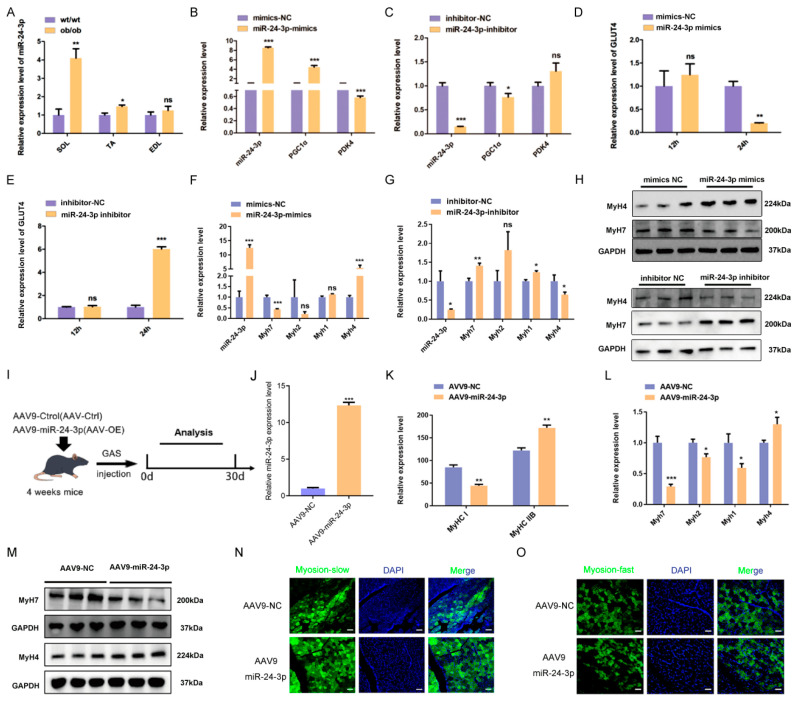
Effects of miR-24-3p on skeletal muscle fiber transformation. (**A**) qRT-PCR was used to analyze miR-24-3p expression in ob/ob mice skeletal muscle. The qRT-PCR analyzed the energy metabolism markers (*PGC1α* and *PDK4*) (**B**,**C**), *GLUT4* (**D**,**E**), and skeletal muscle fiber type transformation marker *(Myh7*, *Myh2*, *Myh1*, and *Myh4*) (**F**,**G**) mRNA expression after overexpression and knockdown of miR-24-3p. (**H**) Western blot was used to analyze skeletal muscle fiber type transformation marker (Myh7 and Myh4) protein expression. (**I**) Diagram of AAV9 virus injection and sample collection patterns. (**J**) qRT-PCR was used to analyze miR-24-3p expression after miR-24-3p was overexpressed by AAV9 in vivo. (**K**–**M**) qRT-PCR and Western blot were used to analyze skeletal muscle fiber type transformation marker expression after miR-24-3p was overexpressed by AAV9 in vivo. (**N**,**O**) Immunofluorescence staining was used to analyze the effect of miR-24-3p overexpression on skeletal muscle fiber type transformation. Scale bar: 50 μm. The data are presented as mean ± S.E.M. and analyzed for statistical differences between groups using unpaired two-tailed *t*-tests. * *p* < 0.05, ** *p* < 0.01, *** *p* < 0.001, ns (not significant).

In our previous study, we reported that the ceRNA network associated with miR-24-3p was enriched in the insulin signaling pathway and played a role in glucose metabolism in skeletal muscle [31]. To further investigate the functional role of miR-24-3p in energy metabolism, we conducted a follow-up study using ob/ob transgenic mice with disturbed energy metabolism due to excessive obesity. We collected SOL, TA, and EDL muscles from ob/ob mice and analyzed the expression of miR-24-3p. The results showed that miR-24-3p expression was higher in ob/ob mice than in wild-type mice (Figure 4A and Figure S7). Moreover, overexpression of miR-24-3p led to an up-regulation of *PGC1α* mRNA expression and a down-regulation of *PDK4* mRNA expression, while knockdown of miR-24-3p resulted in a down-regulation of *PGC1α* mRNA expression (Figure 4B,C). Additionally, changes in the expression of the glucose transporter gene 4 (*GLUT4*) were observed after overexpression and knockdown of miR-24-3p (Figure 4D,E), indicating that miR-24-3p affects skeletal muscle energy metabolism.

Numerous studies have demonstrated that muscle fiber type composition can influence skeletal muscle energy metabolism. Interestingly, the expression of miR-24-3p has been found to be higher in fast-contracting muscles compared to slow-contracting muscles in both pigs and mice (Figure 1E and Figure S1C). This observation led us to hypothesize that miR-24-3p might play a role in influencing metabolism through the regulation of myofiber type conversion. To investigate this further, we conducted experiments using PSMCs and C2C12 myoblasts. The results showed that overexpression of miR-24-3p in PSMCs and C2C12 myoblasts increased the expression of *Myh4*, a slow muscle marker, and decreased the expression of *Myh7*, a fast muscle marker. Conversely, when miR-24-3p was inhibited, the expression of *Myh4* increased while the expression of *Myh7* decreased at both the mRNA and protein levels (Figure 4F–H and Figure S4F).

To further evaluate the effect of miR-24-3p on skeletal muscle fiber type transformation, we conducted in vivo experiments. AAV9-packaged miR-24-3p mimics and mimics NC were injected into the gastrocnemius (GAS) muscle of both legs of mice, with the right leg serving as the control (Figure 4I). Consistent with our in vitro results, qRT-PCR analysis showed a significant overexpression of miR-24-3p in the left leg compared to the control (Figure 4J). Moreover, the overexpression of miR-24-3p resulted in an increase in the expression of *Myh4* and a decrease in the expression of Myh7 (Figure 4K–M). Immunofluorescence staining further confirmed these findings, as there was a significantly increased number of myosin-fast muscle fibers and a decrease in the number of myosin-slow muscle fibers in the GAS muscle after injection of AAV9-miR-24-3p compared to the control group (AAV9-NC) (Figure 4N,O). These experimental results suggest that miR-24-3p is capable of regulating skeletal muscle fiber type transformation.

### 3.5. miR-24-3p Regulates Skeletal Muscle Regeneration

Studies have emphasized the significant impact of myoblast proliferation and differentiation and myofiber type composition on myogenesis. To investigate the molecular elements involved in myogenesis, skeletal muscle regeneration models have been employed to examine the effects of regulatory factors. In our study, we induced skeletal muscle injury by injecting 10 nM of CTX into the TA muscle of C57BL6 mice, using a volume of 20 μL per mouse. We then collected the TA muscle samples at various time points after injury, including 0d, 1d, 3d, 5d, 7d, and 14d, for further examination (Figure S5A). To assess the activation and proliferation of myosatellite cells, we analyzed the expression of *Pax7*, a marker indicative of such processes. The qRT-PCR results revealed high expression levels of *Pax7* during the early stages of skeletal muscle regeneration, suggesting its involvement in the activation and proliferation of myosatellite cells (Figure S4B). Moreover, we examined *MyoD*, *MyoG*, and *MyHC*, which are markers associated with myogenic differentiation. Notably, we observed elevated expression levels of these markers during the late stage of skeletal muscle regeneration, indicating their role in myogenic differentiation (Figure S5B). More importantly, we also noticed changes in miR-24-3p expression during skeletal muscle regeneration. Specifically, miR-24-3p expression was down-regulated in the early stage and up-regulated in the late stage of regeneration, which was opposite to the expression trend of *Pax7* but similar to the expression trends of *MyoD*, *MyoG*, and *MyHC* (Figure S5C). These results provide further evidence of a potential regulatory function for miR-24-3p in myogenesis.

**Figure 5:**
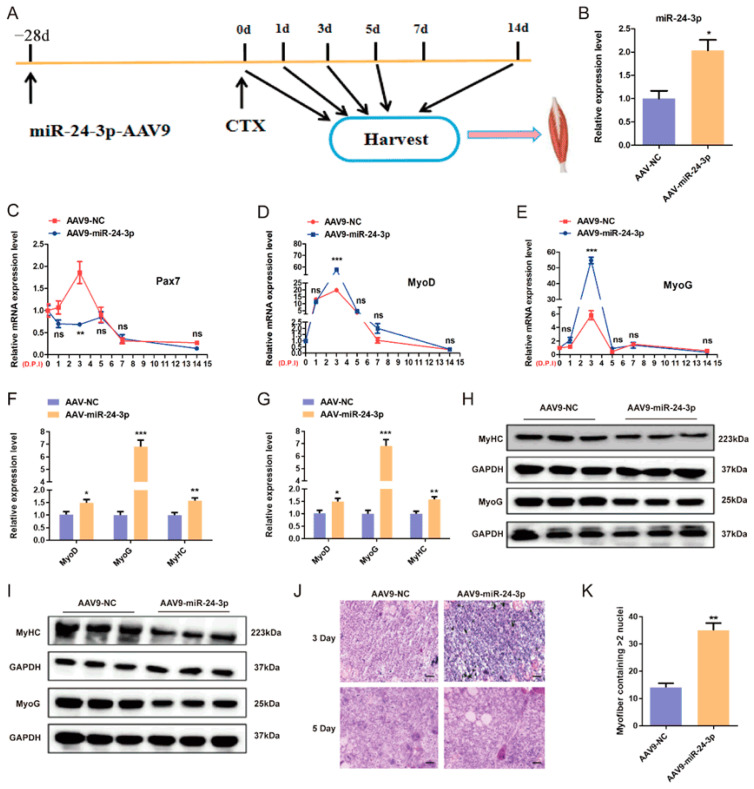
Effects of miR-24-3p on C2C12 myoblast differentiation. (**A**) Schematic representation of the skeletal muscle regeneration pattern constructed after overexpression of miR-24-3p. (**B**) qRT-PCR was used to analyze miR-24-3p expression after miR-24-3p was overexpressed by AAV9 in vivo. (**C**–**E**) qRT-PCR was used to analyze the *Pax7*, *MyoG*, and *MyHC* expressions after miR-24-3p overexpression during skeletal muscle regeneration. (**F**,**G**) qRT-PCR was used to analyze the *MyoD*, *MyoG*, and *MyHC* expressions after miR-24-3p overexpression during skeletal muscle regeneration on day 3 (**F**) and day 5 (**G**). (**H**,**I**) Western blot was used to analyze the MyoG and MyHC expressions after miR-24-3p overexpression during skeletal muscle regeneration on day 3 (**H**) and day 5 (**I**). (**J**) H&E staining results of sections. Scale bar: 50 μm. (**K**) Comparison of the number of newly formed muscle fibers. The data are presented as mean ± S.E.M. and analyzed for statistical differences between groups using unpaired two-tailed *t*-tests. * *p* < 0.05, ** *p* < 0.01, *** *p* < 0.001, ns (not significant).

To investigate the impact of miR-24-3p on skeletal muscle regeneration, we conducted an in vivo experiment using C57BL6 mice. We injected 100 μL of AAV9-packaged miR-24-3p mimics into the TA muscle of the right leg and the same volume of mimics NC into the left leg, aiming to achieve overexpression of miR-24-3p in vivo. After 28 days of AAV9 injection, we induced a skeletal muscle injury by injecting 20 μL of 10 nM CTX into each leg. Subsequently, we collected samples at various time points (0d, 1d, 3d, 5d, 7d, and 14d) during the process of skeletal muscle regeneration to examine the effect of miR-24-3p overexpression (Figure 5A). The results obtained from qRT-PCR showed a highly significant increase in the expression of miR-24-3p. Additionally, we observed a decrease in *Pax7* expression and an increase in *MyoD* and *MyHC* expression (Figure 5B–E).

Furthermore, we compared the effect of miR-24-3p on skeletal muscle regeneration between days 3 and 5. Both qRT-PCR and Western blot results showed that overexpression of miR-24-3p promoted the expression of *MyoD* and *MyHC* on these specific days of skeletal muscle regeneration (Figure 5F–I). Additionally, H&E staining was performed to examine the histological changes in the injured muscles. At day 3, the overexpression of miR-24-3p resulted in an increased number of monocytes and a stronger inflammatory response in the injured muscles, which facilitated the activation of satellite cell proliferation (Figure 5J). By day 5, miR-24-3p overexpression promoted myofiber formation, and a significantly greater number of new myofibers were observed in the TA muscle of the left leg compared to the right leg (Figure 5J,K). Taken together, these results suggest that overexpression of miR-24-3p promotes skeletal muscle regeneration by regulating the expression of genes involved in myogenic differentiation.

### 3.6. The miR-24-3p Target Analysis

**Figure 6:**
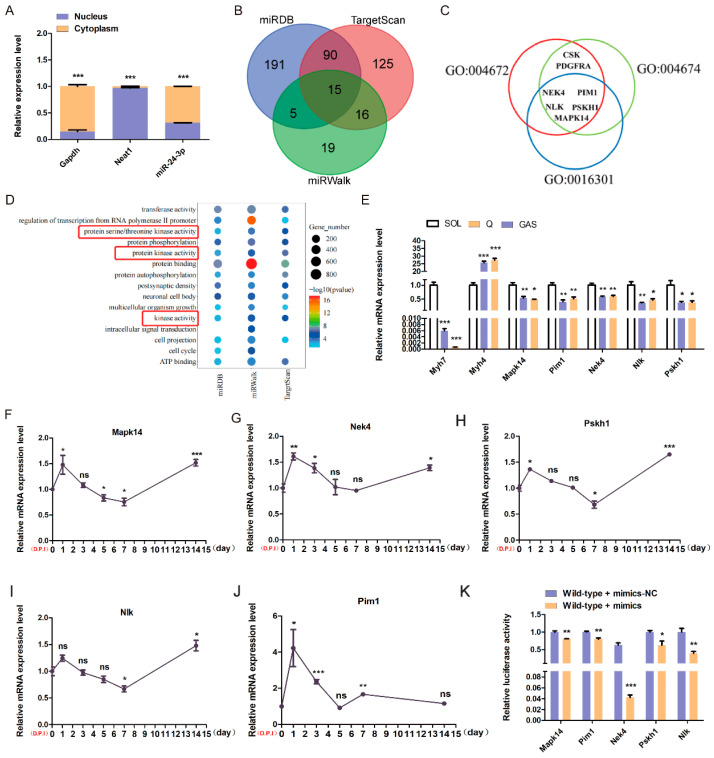
Effects of miR-24-3p on C2C12 myoblast differentiation. (**A**) qRT-PCR was used to analyze miR-24-3p expression in the nucleus and cytoplasm of C2C12 myoblasts. (**B**) Venn plots of the prediction results of miRDB, Target Scan, and miRWalk. (**C**) GO analysis of common predictive target genes in three databases. (**D**) KEGG analysis genes associated with protein kinase activity. (**E**) qRT-PCR was used to analyze gene expression in different types of skeletal muscle. qRT-PCR was used to analyze the expression levels of *Mapk14* (**F**), *Nek4* (**G**), *Pskh1* (**H**), *Nlk* (**I**), and *Pim1* (**J**) during skeletal muscle regeneration in mice. (**K**) Dual luciferase activity detection of miR-24-3p binding to target genes. The data are presented as mean ± S.E.M. and analyzed for statistical differences between groups using unpaired two-tailed *t*-tests. * *p* < 0.05, ** *p* < 0.01, *** *p* < 0.001, ns (not significant).

Finally, we aimed to determine which target genes of miR-24-3p are involved in the regulation of skeletal muscle function. We first investigated the subcellular localization of miR-24-3p and found that it is mainly expressed in the cytoplasm (Figure 6A). Using three online prediction tools (miRDB, TargetScan, and miRWalk), we identified a total of 15 genes that could potentially be targeted by miR-24-3p (Figure 6B). GO analysis revealed five genes (*Mapk14*, *Nek4*, *Pskh1*, *Pim1*, and *Nlk*) were correlated with the protein kinase activity (Figure 6C,D). To further explore the expression patterns of these predicted target genes, we performed qRT-PCR analysis. The results showed that these five genes were generally expressed in various mouse tissues, but their expression levels were relatively lower in skeletal muscle (Figure S6A–E). However, interestingly, the expression levels of *Mapk14*, *Nek4*, *Pskh1*, *Pim1*, and *Nlk* were highly expressed in slow skeletal muscle fiber, which is different from the high expression of miR-24-3p in fast skeletal muscle fiber (Figure 6E). Furthermore, during the injury regeneration process of mouse skeletal muscle, the expression levels of *Mapk14*, *Nek4*, *Pskh1*, *Pim1*, and *Nlk* exhibited a trend of initially increasing and then decreasing, which was contrary to the trend observed for miR-24-3p expression (Figure 6F–K).

Furthermore, we used a dual luciferase assay to confirm whether miR-24-3p directly targets the identified genes. The results demonstrated that miR-24-3p inhibited the activity of the wild-type luciferase vectors containing the binding sites of *Mapk14*, *Nek4*, *Pskh1*, *Pim1*, and *Nlk* while having no effect on the mutant vectors lacking the binding sites (Figure 6K). These experimental results provide further evidence supporting the targeting relationship between miR-24-3p and *Mapk14*, *Nek4*, *Pskh1*, *Pim1*, and *Nlk* in skeletal muscle. Moreover, they suggest that these genes, namely *Mapk14*, *Nek4*, *Pskh1*, *Pim1*, and *Nlk*, may potentially influence mouse skeletal muscle regeneration and myofiber type transformation.

## 4. Discussion

Skeletal muscle plays an important role in the daily life and physical activities of organisms [3,44], and its growth and development directly impact the quality of livestock meat [45]. Abnormal regulation of specific genes in skeletal muscle can lead to various muscle diseases [46]. Skeletal muscle has the ability to regenerate, primarily regulated by four muscle regulatory factors (MRFs) and epigenetic mechanisms. When skeletal muscle is damaged after birth, satellite cells located on the surface of muscle fibers can be activated and differentiate into myoblasts, initiating muscle cell differentiation and forming mature muscle fibers [47,48,49,50]. Skeletal muscle is composed of different types of fibers, mainly classified as slow-twitch and fast-twitch, which possess a wide range of diverse biochemical and metabolic properties. The molecular composition and contractile characteristics of skeletal muscle myofibers dynamically adapt in response to functional demands [51]. The composition of different muscle fiber types is important for the development and maintenance of skeletal muscle [52,53,54].

Numerous studies have demonstrated that miRNA-mediated regulation in skeletal muscle development, particularly in target gene expression and signaling pathway regulation, is particularly important during skeletal muscle development [22]. Our previous RNA-seq analysis of skeletal muscle across 27 developmental stages revealed differential expression of miR-24-3p in Tongcheng and Landrace pigs. This miRNA was found to target 5 lincRNAs and 40 mRNAs closely associated with gluconeogenic processes and insulin signaling pathways [43]. During pig development, somites undergo formation between embryonic days 14 and 22. At embryonic stage E35, primary myotubes are established, and cell proliferation reaches its peak around stage E49. As the primary myotubes continue to proliferate, the formation of secondary myotubes commences, subsequently leading to the disappearance of the primary myotubes. Secondary myotubes undergo proliferation at E90. In the context of secondary myotubes, the number of myofibers is determined prior to birth, while the diameter and length of myofibers progressively increase until D60 after birth [55,56]. In this study, we identified significant differential expression of miR-24-3p during both embryonic (E33–E65) and fetal stages (E70–E105) of skeletal fiber formation. Furthermore, we demonstrated that miR-24-3p influences proliferation, differentiation, and myofiber type transformation. The regulatory function of miR-24-3p in skeletal myogenesis and skeletal muscle fiber transformation was further confirmed in a mouse injury model. These findings suggest that miR-24-3p plays a key role in the growth and development of porcine skeletal muscle and is a potential regulator of meat yield and quality in pigs, making it a promising target for future improvements in pig breeds, pork yield, and meat quality.

miRNAs exhibit in-sequence and functional conservation across species. In mice, miR-378 targets the degradation of *MyoG*, enhances the transcriptional activity of *MyoD*, promotes the differentiation of C2C12 cells, and regulates the regeneration of mouse skeletal muscle [57]. Additionally, miR-378 has been found to promote differentiation and myotube fusion in bovine skeletal muscle satellite cells [58]. Both mice and cattle have shown that overexpression of miR-1/206 targets *PAX7* and regulates the proliferation and myogenic differentiation of bovine skeletal muscle cells [59]. In our study, miR-24-3p was up-regulated during the differentiation of PSMCs and C2C12 myoblasts. Further functional experiments revealed that overexpression of miR-24-3p promotes differentiation and inhibits proliferation in PSMCs and C2C12 myoblasts. Moreover, the regulatory role of miR-24-3p in the transformation of skeletal muscle fiber types was consistent in both porcine and murine skeletal muscle. Therefore, our study demonstrates that miR-24-3p is an important and functionally conserved miRNA across different species, playing a regulatory role in various life processes within the animal body.

Protein kinase catalysis is a fundamental biochemical reaction in cells of significant importance. Among the five major classes of protein kinases, including histidine protein kinases and aspartate/glutamyl protein kinases, phosphorylation and dephosphorylation mediated by these kinases play crucial roles in signal transduction and important biological processes in eukaryotic cells [60]. Adenosine monophosphate-activated protein kinase, known as *AMPK*, is essential for myocyte energy metabolism, proliferation, and differentiation [61,62]. Studies have shown that in rodents, exercise and muscle contraction increase *AMPKα2* activity [63]. Activation of *AMPK* using drugs such as metformin inhibits the differentiation of C2C12 cells, while the absence of *AMPK* in skeletal muscle satellite cells hinders regeneration after skeletal muscle injury [64]. Furthermore, protein kinase D1 *(PKD1)* is highly expressed in type I muscle fibers and serves as a key regulator of skeletal muscle function and phenotype by promoting the transcription and expression of *MEF2*, which induces the conversion of type II muscle fibers to type I and enhances skeletal muscle endurance [65]. These findings suggest that protein kinases play vital regulatory roles in the growth and development of skeletal muscle. In our study, we identified and validated five target genes of miR-24-3p, namely *Mapk14*, *Nek4*, *Pskh1*, *Pim1*, and *Nlk*, which are protein kinases, in mice and pigs. We observed higher expression levels of *Mapk14*, *Nek4*, *Pskh1*, *Pim1*, and *Nlk* in pig and mouse slow muscles compared to fast muscles, indicating potential involvement in the transformation of skeletal muscle fiber types. Additionally, in a mouse model of skeletal muscle regeneration, the expression levels of *Mapk14*, *Nek4*, *Pskh1*, *Pim1*, and *Nlk* showed an increasing and then decreasing trend with the activation of myosatellite cells for proliferation and the formation of new myofibers. These results suggest that miR-24-3p may regulate skeletal muscle fiber type transformation by binding to these gene clusters (*Mapk14*, *Nek4*, *Pskh1*, *Pim1*, and *Nlk*). Furthermore, it has been shown that many genes are involved in skeletal muscle regeneration and myofiber type transformation, such as MEETL8 and SOX6 inhibiting slow myogenesis by suppressing MYH7 expression [66,67], and Map3k20 and LSD1 playing important roles in regulating skeletal muscle regeneration [68,69]. We speculate that miR-24-3p may also target these genes in regulating myofiber type transformation and skeletal muscle regeneration. However, further investigation is required to determine whether miR-24-3p can regulate skeletal muscle cell proliferation, differentiation, and myofiber type transformation through these targets.

## 5. Conclusions

In this study, we analyzed differentially expressed miRNAs by analyzing different periods of porcine skeletal muscle development and found that miR-24-3p was significantly different in both embryonic (E33–E65) and fetal (E70–E105) stages and had higher expression in Tongcheng (TC) pigs than in Landrace (LD) pigs. Further studies have shown that miR-24-3p inhibits skeletal muscle cell proliferation, promotes differentiation, and regulates skeletal muscle fiber transformation in pigs and mice. Moreover, we found miR-24-3p overexpression promotes skeletal muscle regeneration and fast-myofiber myogenesis in vivo. In addition, in this study, we performed a preliminary exploration of the target genes of miR-24-3p and found that miR-24-3p was able to affect the dual luciferase reporter vector activity of the *Nek4*, *Pim1*, *Nlk*, *Pskh1*, and *Mapk14* genes. These results suggest that miR-24-3p may affect myogenesis and skeletal muscle fiber type transformation by regulating the expression of different genes.

## Data Availability

No new data were created or analyzed in this study. Data sharing is not applicable to this article.

## Supplementary Materials

The following supporting information can be downloaded at: https://www.mdpi.com/article/10.3390/genes15030269/s1, Figure S1: miR-24-3p expression pattern in skeletal muscle; Figure S2 Effect of miR-24-3p on C2C12 myoblasts differentiation; Figure S3 conservation analysis of miR-24-3p sequence in different species; Figure S4 Effect of miR-24-3p on skeletal muscle fiber type transformation in pig; Figure S5 The miR-24-3p expression during skeletal muscle regeneration; Figure S6 The effects of ssc-miR-24-3p on proliferation marker genes of porcine skeletal muscle primary cells, Figure S7: Fast and slow muscle expression in wild mice and ob/ob mice; Table S1: Primer sequences for reverse transcription and qPCR.
