# Supplementary material for: Regulation of myo-miR-24-3p on the Myogenesis and Fiber Type Transformation of Skeletal Muscle
Source: Genes (Basel). 2024 Feb 21;15(3):269. doi: 10.3390/genes15030269 (PMC10970682; doi:10.3390/genes15030269)
Supplement: Supplementary file 1 [file genes-15-00269-s001.zip › genes-2833129-supplementary.pdf]

Supplementary information

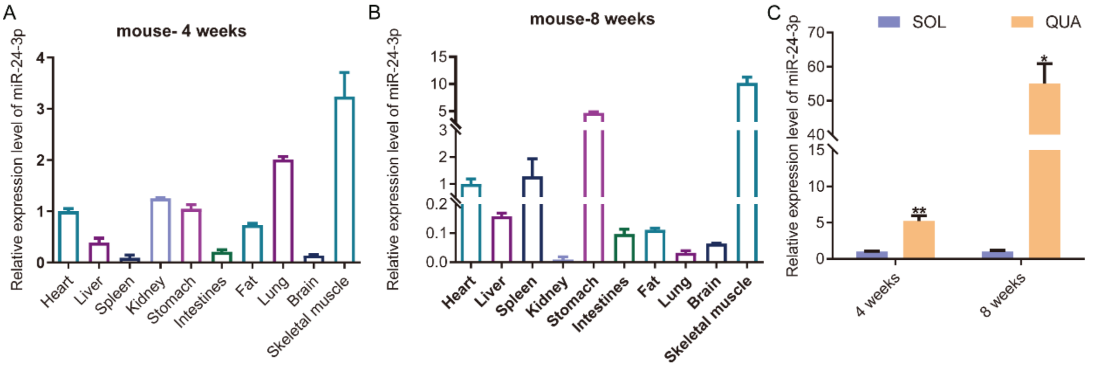

Figure S1 miR-24-3p expression pattern in skeletal muscle

(A-B) miR-24-3p expression in different tissues at weeks 4 and 8 of mouse development. (C) miR-24-3p expression in fast and slow muscles at weeks 4 and 8 of mouse development. Data are presented as mean ± S.E.M and analyzed for statistical differences between groups using unpaired two-tailed t-tests. *$p < 0.05$, **$p < 0.01$.

Table S1 Primer sequences for reverse transcription and qPCR

Name	Sequence(5’-3’)
miR-24-3p	RT: GTCGTATCCAGTGCAGGGTCCGAGGTATTCG CACTGGATACGACCTGTTC
	F: GCGTGGCTCAGTTCAGCAG
	R：GCCCTGCATCTTGAAATCTGA
Pig-U6	F: CTCGCTTCGGCAGCACA
	R: AACGCTTCACGAATTTGCGT
Pig-β-ACTIN	F: GCGGCATCCACGAAACTAC
	R: TGATCTCCTTCTGCATCCTGTC
Pig-GAPDH	F: AGGTCGGTGTGAACGGATTTG
	R: TGTAGACCATGTAGTTGAGGTCA
Pig-NEAT	F: GTCGATGCCCTGAACATG
	R: GGTTAGCACGGAACCTACA
Pig-KI67	F: AGCCCGTATCTGGTGCAAAA
	R: CCTGCATCTGTGTAAGGGCA
Pig-PCNA	F: AAGTCAAATCTGGTCTTGTTAGCC
	R: CACTGTCCTGGGATGCTTGAA
Pig-CDK4	F: TGGTTACAAGTGGTGGGACA
	R: CTGGAGCACGGTACCAGAGT

Pig-CYCLIN A	F: GCAGCAGCCTTTCATTTAGC
	R: GGTGAAGGTCCAGGAGACAA
Pig-CYCLIN D2	F: GTGCTGGGCAAGTTGAAGTG
	R: GCGAACTTGAAGTCAGTGGC
Pig-MyoD	F: ATGAGACATCCCCCTACTTCTACCA
	R: GTCCCCAGCCCCTTATCTTCC
Pig-MyoG	F: CCTGCTCAGCTCCCTCAAC
	R: CACAGCCACATCCTCCACT
Pig-MyHC	F: GTTCAGAGAAAGGCATCCCAAA
	R: GAGAGTGACCGACACCACAAGTG
Pig-MYH7	F: AAGGGCTTGAACGAGGAGTAGA
	R: TTATTCTGCTTCCTCCAAAGGG
Pig-MYH4	F: ATGAAGAGGAACCACATTA
	R: TTATTGCCTCAGTAGCTTG
Pig-MYH1	F: AGAAGATCAACTGAGTGAACT
	R: AGAGCTGAGAAACTAACGTG
Pig-MAPK14	F: CTACAGAGAACTGCGGTTACT
	R: GTAAGCTTCTGACATTTCACAA
Pig-NLK	F: TGGATATTGAGCCGGATAGA
	R: GCAAGAGACCAGATTCTGGAA
Pig-NEK4	F: CAATTAGATGCCTCTAATGAGCT
	R: CTCTAGTAATATTGTCAGAGG
Pig-PIM1	F: GCAAGACCTCTTCGACTTTATC
	R: CGATGAGGATGTTCTCGTCCTT
Pig-PSKH1	F: CATCATCGCCAAGGGTTCTTTCA
	R: TGCCTGGATGGTAGTAGAGCA
Mouse-U6	F: CTCGCTTCGGCAGCACA
	R: AACGCTTCACGAATTTGCGT
Mouse-Gapdh	F: CCTGTTGCTGTAGCCGTATT
	R: CATCAAGAAGGTGGTGAAGC
Mouse-Pax7	F: CCTGGAACAGACAGAGAGGAGCAGGAGAG
	R: GTGAGTTCCTTCACTCTGCGCTCGTGC
Mouse-Ki67	F: TGCCCGACCCTACAAAATG
	R:GAGCCTGTATCACTCATCTGC
Mouse-Pcna	F: GGGTGAAGTTTTCTGCAAGTG
	R: GTACCTCAGAGCAAACGTTAGG
Mouse-Cdk4	F: ACAAGTAATGGGACCGTCAAG
	R: GGGTGTTGCGTATGTAGACTG

Mouse-Cyclin D1	F: GCCCTCCGTATCTTACTTCAAG
	R: GCGGTCCAGGTAGTTCATG
Mouse-Cyclin E1	F: GCGAGGATGAGAGCAGTTC
	R: AAGTCCTGTGCCAAGTAGAAC
Mouse-MyOD	F: CCAGCACTACATTTGGCGACTAAT
	R: GCTCCAATATGCTGGACAGGCAGT
Mouse-MyOG	F: GCCCAGTGAATGCAACTCCCACA
	R: CAGCCGCGAGCAAATGATCTCCT
Mouse-MyHC	F: ACAAGCTGCAGCTGAAGGTG
	R: TCATTCAGGCCCTTGGCAC
Mouse-Myh7	F: ACAAGCTGCAGCTGAAGGTG
	R: TCATTCAGGCCCTTGGCAC
Mouse-Myh1	F: TGCAACAGTTCTTCAACCAC
	R: GCCAGGTCCATCCCAAAGT
Mouse-Myh2	F: CCAGCTGCACCTTCTCGTTTGCCAG
	R: CATGGGGAAGATCTGGTCTTCTT
Mouse-Myh4	F: CCTGGAACAGACAGAGAGGAGCAGGAGAG
	R: GTGAGTTCCTTCACTCTGCGCTCGTGC
Mouse-Mapk14	F: GTTTCTCATCTACCAGATCCT
	R: TAGCCTGTCATCTCATCATCA
Mouse-Nlk	F: GAGTCTTCCGGGAATTGAAGAT
	R: AGATCACTCTGCATCAATTCT
Mouse-Nek4	F: ATTACATGAGCCCTGAGCTGTT
	R: GTGCTGTAAACTTTTGGCATT
Mouse-Pim1	F: TTCGGCTCGGTCTACTCTGGCAT
	R: ACCTTCTTCAACAGGACCA
Mouse-Pskh1	F:TATCAAGATGATAGAGACCA
	R:AGCTCCATCACCATATATACAC

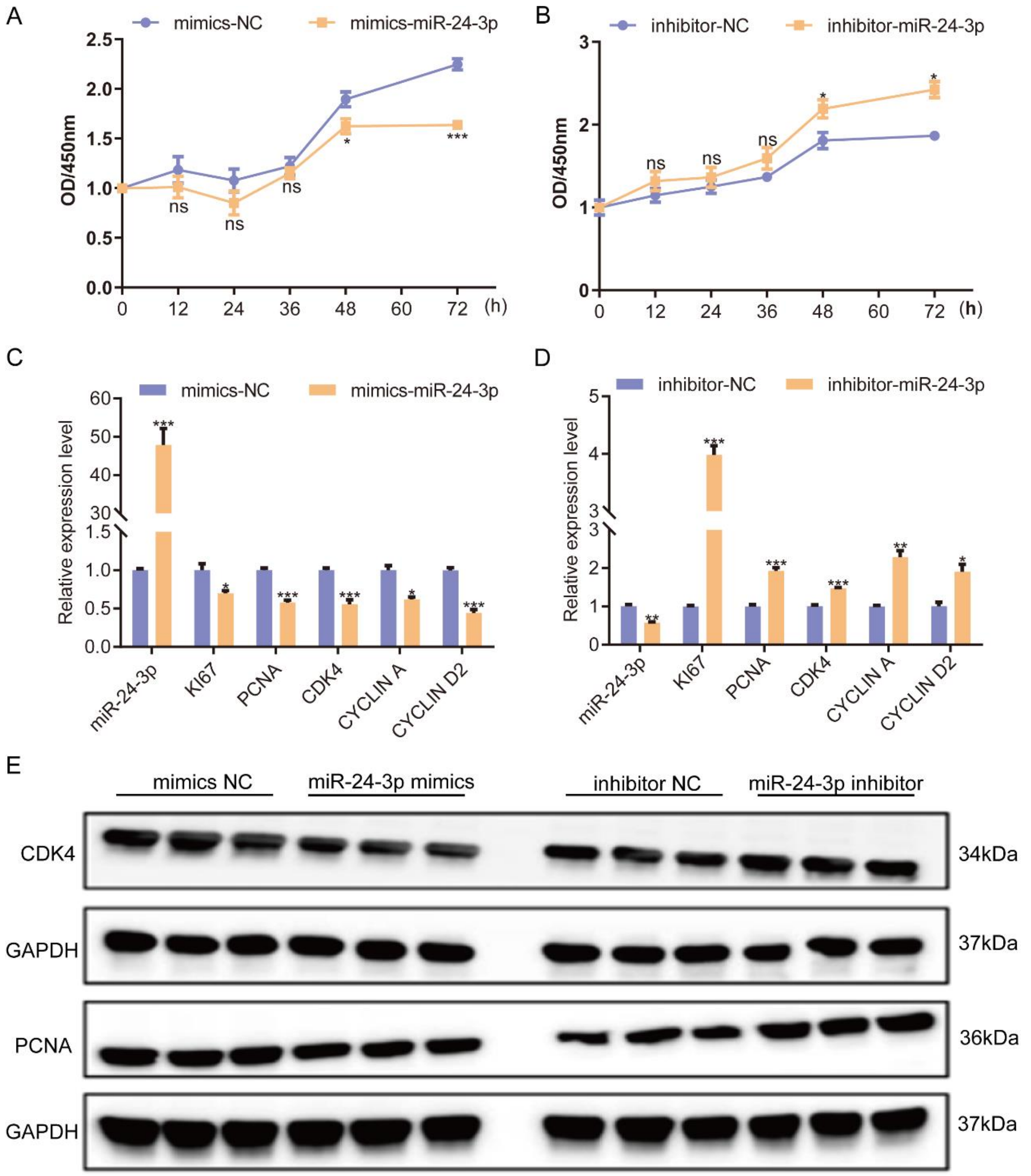

Figure S2 Effect of miR-24-3p on C2C12 myoblasts differentiation

(A-B) CCK-8 analyzed cell proliferation after miR-24-3p overexpression and knockdown in PSMCs. (C-D) qRT-PCR analyzed cell proliferation markers (KI67, PCNA, CDK4, CYCLINA and CYCLIND2) mRNA expression after miR-24-3p overexpression and knockdown in PSMCs. (E) Western blot analyzed proliferation markers (CDK4 and PCNA) protein expression after miR-24-3p overexpression and knockdown in PSMCs. Data are presented as mean ± S.E.M and analyzed for statistical differences between groups using unpaired two-tailed t-tests. **p* < 0.05, ***p* < 0.01, ****p* < 0.001, *ns* (not significant).

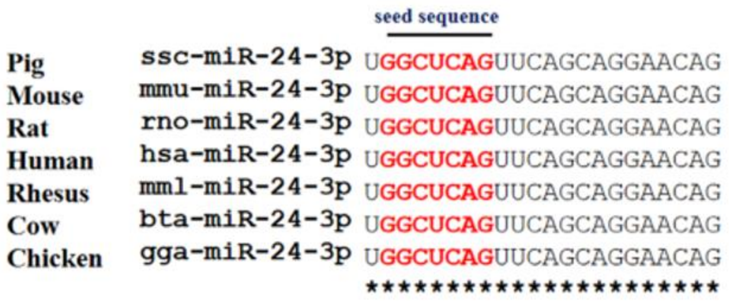

Figure S3 conservation analysis of miR-24-3p sequence in different species

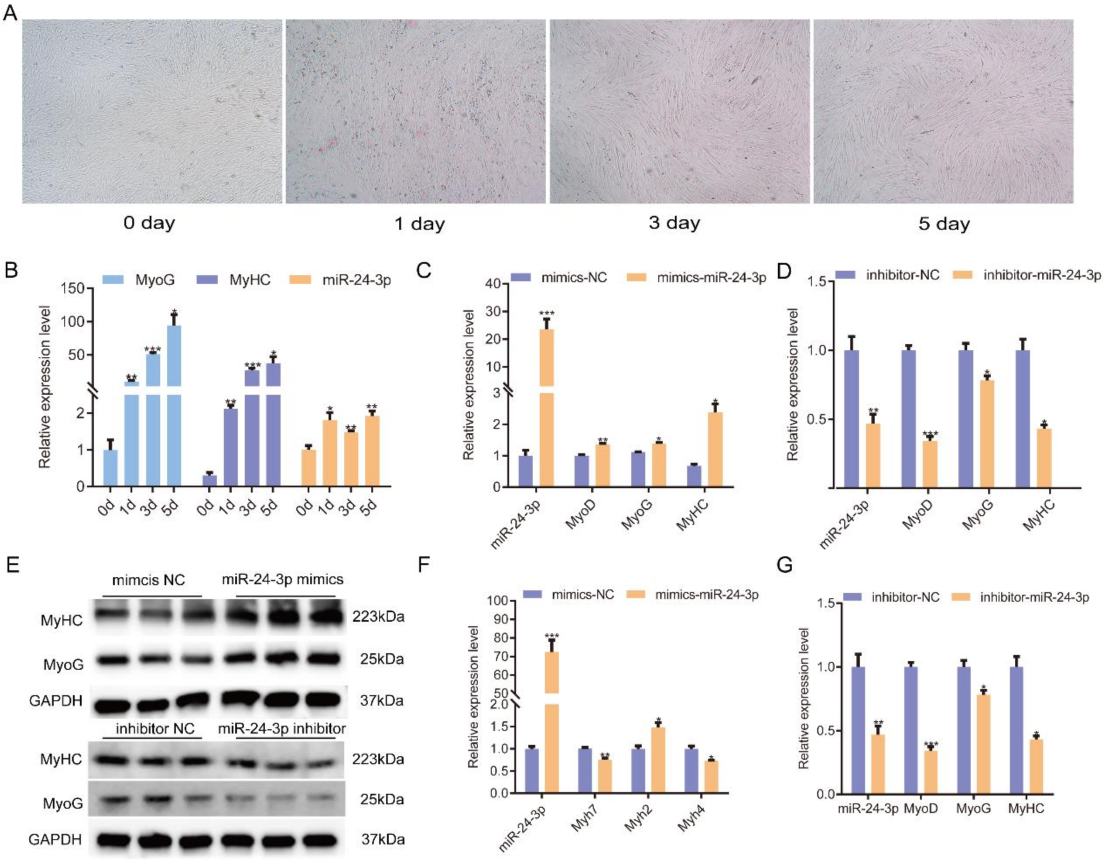

Figure S4 Effect of miR-24-3p on skeletal muscle fiber type transformation in pig

(A) PSMCs were induced to differentiate for different days (0d, 1d, 3d and 5d). Scale bar: 50 μm. (B) qRT-PCR analyzed the expression pattern of MyoG, MyHC and miR-24-3p during PSMCs differentiation. (C-D) qRT-PCR analyzed the expression of miR-24-3p, MyoG, MyHC and MyoD after miR-24-3p overexpression and knockdown. (E) Western blot analyzed the protein expression of MyoG and MyHC after miR-24-3p overexpression and knockdown. (F-G) qRT-PCR analyzed the expression of differentiation and transformation markers expression after miR-24-3p overexpression and knockdown. Data are presented as mean ± S.E.M and analyzed for statistical differences between groups using unpaired two-tailed t-tests. *$p < 0.05$, **$p < 0.01$, ***$p < 0.001$.

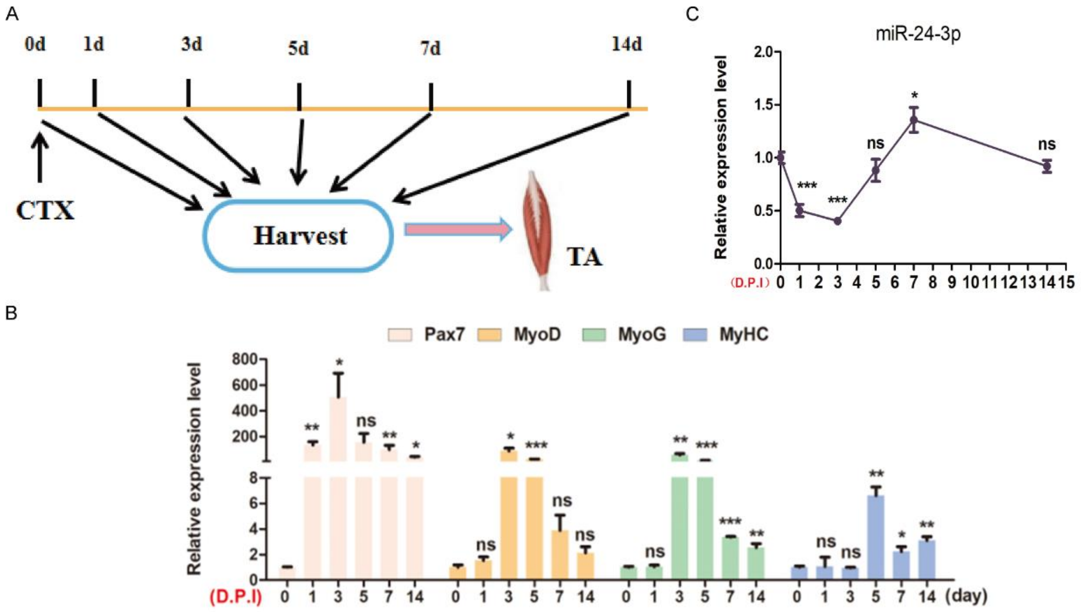

Figure S5 The miR-24-3p expression during skeletal muscle regeneration

(A) Schematic representation of the skeletal muscle regeneration. (B-C) qRT-PCR analyzed the expression pattern of Pax7, MyoD, MyoG, MyHC and miR-24-3p during skeletal muscle regeneration. Data are presented as mean ± S.E.M and analyzed for statistical differences between groups using unpaired two-tailed t-tests. *$p < 0.05$, **$p < 0.01$, ***$p < 0.001$, *ns* (not significant)

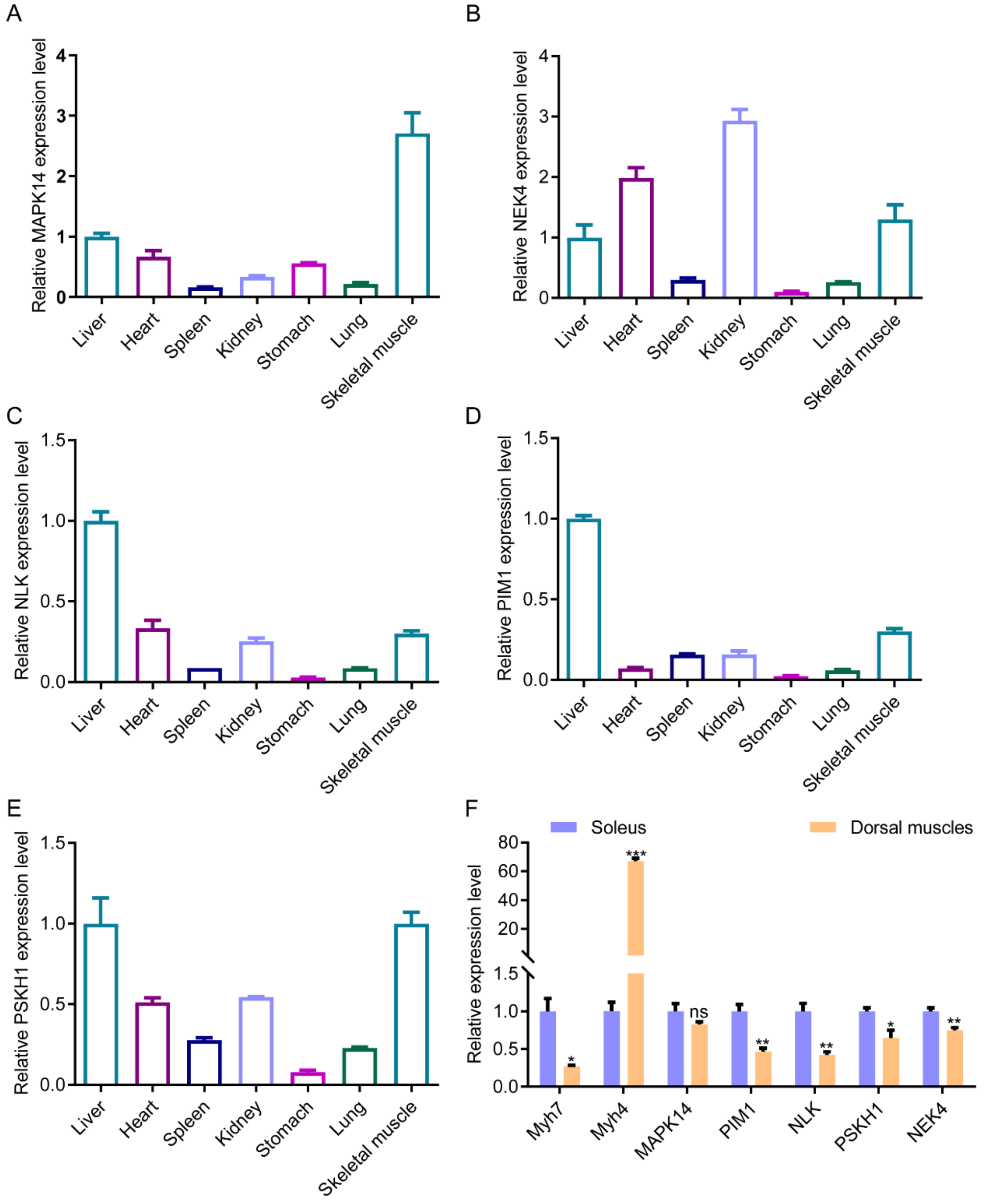

Figure S6 The effects of ssc-miR-24-3p on proliferation marker genes of porcine skeletal muscle primary cells

The expression levels of MAPK14 (A), NEK4 (B), PSKH1 (C), PIM1 (D) and NLK (E) in pig tissues. (F) Expression levels of potential target genes of ssc-miR-24-3p in pig fast and slow muscle. Data are presented as mean ± S.E.M and analyzed for statistical differences between groups using unpaired two-tailed t-tests. **p* < 0.05, ***p* < 0.01, ****p* < 0.001, *ns* (not significant).

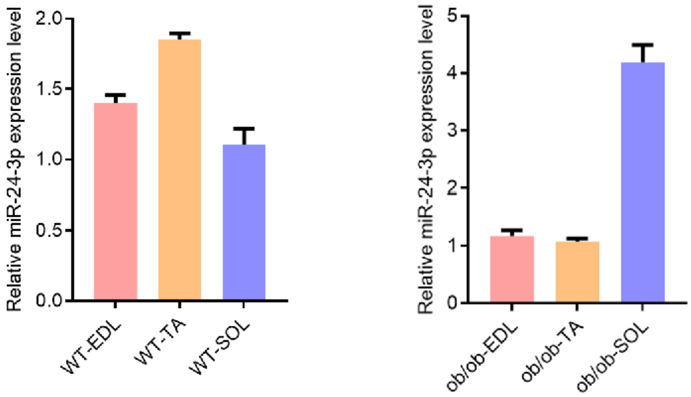

Figure S7: Fast and slow muscle expression in wild mice and ob/ob mice.
